# Intercalary reconstruction of long bones by massive allograft: Comparison of construct stability ensured by three different host-graft junctions and two types of fixations in a synthetic femur model

**DOI:** 10.3389/fped.2022.868299

**Published:** 2022-08-03

**Authors:** Massimiliano Baleani, Paolo Erani, Manon Blaise, Roberta Fognani, Marco Palmas, Marco Manfrini

**Affiliations:** ^1^Laboratorio di Tecnologia Medica, IRCCS Istituto Ortopedico Rizzoli, Bologna, Italy; ^2^Clinica Ortopedica e Traumatologica III a Prevalente Indirizzo Oncologico, IRCCS Istituto Ortopedico Rizzoli, Bologna, Italy

**Keywords:** bone tumours, intercalary allograft reconstruction, host-graft interface, biomechanical behaviour, reconstruction stability

## Abstract

An intercalary segmental allograft is an option for limb salvage in bone tumours. Stable and congruent intercalary reconstructions are a prerequisite for achieving host-graft union. However, a too rigid fixation could increase the risk of late complications correlated with negative bone remodelling. This study compared the reconstruction stiffness achieved by three different host-graft junctions, namely, end-to-end, modified step-cut, and taper. A low-stiffness bone plate was used as the fixation method, except for the taper junction where a low-stiffness intramedullary nail was also used to investigate the effects of different types of fixation on construct stiffness. Composite femora were tested under four loading conditions to determine coronal and sagittal bending stiffness, as well as torsional stiffness in opposite directions. Stiffness values were expressed as a percentage of intact host bone stiffness (%IBS). While a reduction of coronal bending stiffness was found with taper junctions (76%IBS) compared with the high values ensured by end-to-end (96%IBS) and modified step-cut junctions (92%IBS), taper junctions significantly increased stiffness under sagittal bending and torsion in intra- and extra-direction: end-to-end 29%IBS, 7%IBS, 7%IBS, modified step-cut 38%IBS, 20%IBS, 21%IBS, and taper junction 52%IBS, 55%IBS, 56%IBS, respectively. Construct stiffness with taper junctions was decreased by 11–41%IBS by replacing the bone plate with an intramedullary nail. Taper junctions can be an alternative to achieve intercalary reconstructions with more homogeneous and, in three out of four loading conditions, significantly higher construct stability without increasing bone plate stiffness. The risk of instability under high torsional loads increases when taper junctions are associated with a low-stiffness intramedullary nail.

## Introduction

Intercalary reconstruction surgery allows limb preservation after the removal of bone tumours located in long-bone diaphysis and metaphysis. Among the reconstructive options, surgeons can choose to perform an intercalary massive allograft, possibly combined with a vascularised fibular autograft (1, 2).

Besides infection, the two other major complications associated with an intercalary massive allograft are non-union and late intercalary reconstruction fracture (2–5).

In general, patients’ habits and health status have a significant impact on the risk for non-union (6–11). For oncological patients, adjuvant therapies may also impact negatively the rate of non-union (12–14). Nevertheless, the mechanical stability of intercalary reconstruction remains a prerequisite for achieving host-graft union (15, 16). Moreover, a gap at the host-graft interface delays, or even jeopardises, the healing process (17). Therefore, a stable and congruent intercalary reconstruction, with maximum contact between the graft and viable host bone tissue, is vital (17, 18).

Late intercalary reconstruction fractures have been observed in physically active patients or due to local stress concentration in the allograft area (15, 19–22).

The use of strong fixation devices has been advocated to ensure successful postoperative recovery. A strong fixation device not only allows early weight-bearing but also reduces the risk of intercalary reconstruction fracture in short- and mid-term follow-up (23). However, an excessively strong fixation device, which induces stress-shielding within the intercalary reconstruction, leads to a negative bone remodelling balance in the long term (24). The impact of stress-shielding on different biological and biomechanical factors cannot be excluded *a priori*. Since this might contribute to the occurrence of intercalary reconstruction fracture occurring in long-term follow-up (25), stress-shielding within the intercalary reconstruction should be minimised, especially in young patients, since their bone segments are still undergoing active remodelling (26).

In young patients, a telescopic junction for the host-graft junction has been proposed instead of the end-to-end or step-cut junctions (27) in order to (i) achieve a congruent and mechanically stable, i.e., stiff, reconstruction and (ii) minimise cortical atrophy by reducing stress-shielding with the use of less strong fixation. We proposed the use of conical reamers, achieving a taper junction, to facilitate and ensure proper tapering between the host bone and graft ends. The taper junction is an evolution of the concave-convex junction – where the host-graft interface is spherical (28) – that provides an inherent stable connection while still ensuring a large tight contact between the graft and viable host bone tissue. However, the potential advantages of this solution, in terms of mechanical stability of the intercalary reconstruction under different loading conditions, have not been investigated yet. Therefore, this proof-of-concept study compared:

(i) The bending and torsional stability of intercalary reconstructions with three different host-graft junctions, namely, end-to-end, step-cut, and taper. All intercalary reconstructions were fixed with a low-stiffness bone plate [unicortical fixation (29)], to determine the contribution of their junction shapes to the construct stiffness.

(ii) The bending and torsional stability of an intercalary reconstruction fixed with two different types of fixation, namely, low-stiffness bone plate and intramedullary nail. Both intercalary reconstructions included taper junctions to evaluate the effects of the types of fixation on the construct stiffness.

## Materials and methods

### Composite femora and fixation devices

All intercalary reconstructions were performed using composite femora, which are suitable for comparative biomechanical studies. A medium-size femur (length 455 mm, average diaphyseal diameter 27 mm, Sawbones Europe AB, Malmö, Sweden) was chosen as a model of the diseased femur, hereinafter referred to as “host bone”, of a 15-year-old boy. This model was selected using (i) the 50th percentile for height equal to 170 cm (30) and (ii) the relationship between subject height and femur length determined for Caucasian males [height (cm) = 2.32 × femur length (cm) + 65.53 (cm), (31)]. A large-size femur (length 485 mm, average diaphyseal diameter 30 mm, Sawbones Europe AB, Malmö, Sweden) was chosen as the “donor segment” from which the massive graft was extracted. This approach ensured a difference in the cross-section dimensions of the massive graft and host bone to replicate the mismatch surgeons find in the clinical scenario.

A reference system was marked on all the femora prior to osteotomy (32, 33). The sagittal and coronal planes, their intersection line (longitudinal axis of the femur), and the midpoint of the biomechanical length of the femur were used to secure the reproducibility of each step of the experimental procedure.

Two types of fixation devices were used to stabilise the intercalary reconstruction, namely, bone plate and intramedullary nail. Low stiffness implants (Bone plate: LCP 4.5/5.0 mm narrow, 10 holes, centre-to-centre hole distance 18 mm, length 188 mm, TiCP, Synthes GmbH, Switzerland; Intramedullary nail: Dynamic T Femur, left, diameter 10 mm, left length 370 mm, titanium, Citieffe, Italy) were used to decrease the mechanical contribution of the fixation device and, at the same time, enhance the contribution of the shape of the two host-graft junctions to the intercalary reconstruction stiffness.

### Femoral diaphysis resection and massive graft extraction

**FIGURE 1:**
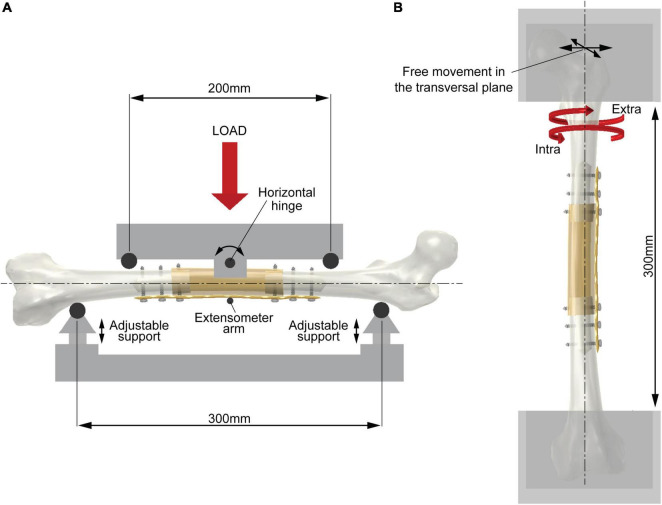
Scheme of the experimental setup. **(A)** Four-point bending test (showing bending in the coronal plane). The two support rollers (span 300 mm) are adjustable in the vertical direction to place the femoral axis in the horizontal position. The two loading rollers (span 200 mm) rotate around a horizontal hinge to ensure equal force between the roller and diaphysis surface during testing. An extensometer measures the vertical deflection of the mid cross-section. **(B)** Torsion test. The femoral axis is aligned with the testing machine axis. The two epiphyses are constrained by acrylic resin in metal holders leaving a free length of 300 mm. The upper holder is free to move in the transversal plane. The biaxial testing machine avoids undesired axial load during testing.

The length of diaphysis resection in the host bone was determined by considering that the whole intercalary reconstruction had to undergo uniform bending and torsional moment. The longitudinal length of the resection had to be shorter than the distance between the inner loading rollers (200 mm, Figure 1A) in the four-point bending test as well as the free length between the edges of the two clamps in the torsion test (300 mm, Figure 1B).

Additionally, the longitudinal length of diaphysis resection had to be a multiple of the centre-to-centre distance between holes of the bone plate to achieve a 9-mm longitudinal shift of the screw axis with respect to the two diaphyseal osteotomies. To satisfy these requirements, the length of diaphysis resection was set at 72 mm. Therefore, the two osteotomies were performed 36 mm apart from the midpoint of the biomechanical length of all host bones.

**FIGURE 2:**
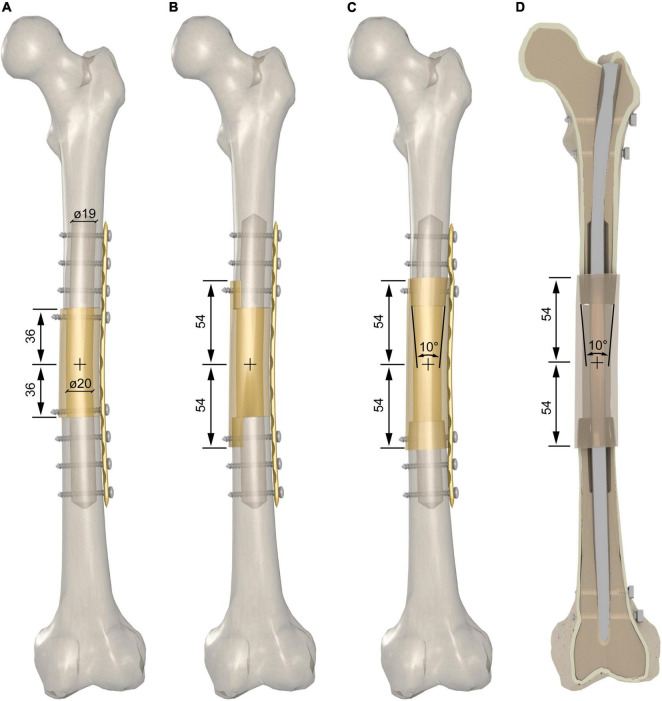
**(A)** End-to-end junction reconstruction with bone plate fixation. Two additional cortex screws were added to fix the massive graft. **(B)** Modified step-cut junction reconstruction with bone plate fixation. **(C)** Taper junction reconstructions with bone plate fixation; **(D)** taper junction reconstructions with intramedullary nail fixation.

The massive graft was extracted from the diaphysis of 12 donor segments. Two osteotomies were performed 36 mm apart from the midpoint of the biomechanical length to extract the grafts for the intercalary reconstruction with the end-to-end junctions. The aforementioned distance was increased to 54 mm to provide an 18-mm overlap of the massive graft for intercalary reconstruction with the step-cut or taper junctions on each side (Figures 2A–D). Prior to proceeding with the simulation of the host bone reconstruction, we decreased the cortical wall of the host bone and massive graft down to 3.5–4.5 mm – values that fall within the lower range of diaphyseal cortical thickness in teens (34) – and 4.5–5.5 mm – values that fall within the lower range of diaphyseal cortical thickness in adults (35), respectively. This was achieved by drilling the medullary canal of the host bone and the massive graft with a 19- and 20-mm drill bit, respectively (Figures 2A–D).

### Intercalary reconstruction techniques

We investigated three different intercalary reconstruction techniques. They differed in the shape of the following two host-graft junctions: (i) two end-to-end junctions, achieved by cutting both the host bone and the graft orthogonally to the longitudinal axis (Figure 2A) and (ii) two modified step-cut junctions, achieved by cutting the host bone orthogonally to the longitudinal axis while an 18 mm step-cut was created in a parasagittal plane on the graft, i.e., a plane shifted 13 mm medial to the sagittal plane (Figure 2B). The 18-mm step was cylindrically reamed to 26-mm diameter on the inner side achieving a cortical shell with an inner cylindrical surface. The medial side of the host bone was also contoured to a 26-mm diameter to achieve a perfect match with the cortical shell of the graft; (iii) two taper junctions were achieved by cutting the host bone orthogonally to the longitudinal axis and tapering the two ends by means of a milling cutter (Figures 2C,D). The taper angle was set at 10° to avoid excessive thinning of the cortex of the host bone along the 18 mm longitudinal length of the taper. A female tapered cavity was reamed out on both ends of the graft. This angle was also set at 10° to achieve a perfect match with the male tapers created on both host bone ends.

The reference system marked on each femur was used to align the two host bone stumps and the massive graft properly and to restore the anteroversion angle of the femoral neck. The host bone stumps were kept in the desired spatial position, i.e., aligned with the lateral side oriented upward to make plate or nail fixation easier, by means of two-part rigid moulds clamping the proximal and distal epiphysis. The massive graft was medially supported, except for those with taper junction that was self-sustaining once assembled.

Bridging plate or nail fixation was performed following the operative technique provided by the manufacturer.

The bone plate was placed on the lateral side of the diaphysis with the midplate aligned with the midpoint of the massive graft. It was not necessary to bend the plate to match the lateral contour of the intercalary reconstruction. Starting from the most proximal and distal hole and proceeding toward the middle of the plate, (i) a 3.2-mm hole was drilled using the LCP drill guide in a neutral position except for the most proximal hole, which was instead drilled with the drill guide in eccentric position to achieve dynamic compression; (ii) the screw length was determined using a depth gauge; (iii) the hole was tapped for a 4.5-mm cortex screw; (iv) the screw of appropriate length was manually inserted and tightened to 5 Nm. Screws were inserted on the three most proximal and distal holes of the plate (Figures 2B,C), except for the end-to-end junction where two additional screws were inserted to fix the massive graft, i.e., screws were inserted on the four most proximal and distal holes of the plate (Figure 2A).

The intramedullary nail was inserted on the great trochanter. A 10-mm hole was drilled on the tip of the great trochanter and manually reamed using the specific tool. The inner part of the distal stump was reamed using an 11-mm flexible reamer, i.e., 1 mm larger than the diameter of the nail. Following nail insertion, the lateral guide was used to drill a transversal pre-hole and insert the most proximal 5-mm self-tapping screw. The nail was locked distally with two transversal 5-mm self-tapping screws inserted after having drilled two pre-holes using the targetting device. Slightly longitudinal compaction was achieved by tightening the proximal axial screw to 3 Nm. Finally, a second proximal hole was drilled using the targetting device, and the second proximal 5-mm self-tapping screw was inserted (Figure 2D).

### Mechanical characterisation of intercalary reconstruction technique

Bending and torsion testing was performed to determine the stiffness of the 12 intercalary reconstructions and three intact host bones, whose average value was used as a reference to express the stiffness of each intercalary reconstruction as a percentage of intact host bone stiffness (%IBS).

#### Bending stiffness

All femora were tested under four-point loading conditions. The support and loading spans were 300 and 200 mm, respectively (Figure 1A). The height of the support rollers was adjustable to allow horizontal alignment of the femoral axis. The loading rollers were free to rotate halfway around a horizontal axis to follow any asymmetrical deformation of the reconstruction and ensure equal force values on the two loading rollers. Before testing, the lateral and anterior sides of each host bone were flattened, at the contact area with the support rollers, by milling the femur surface for a longitudinal length of 10 mm to ensure perfect contact and the desired circumferential orientation of the femur.

A preload of 0.05 kN was applied to the femur using a material testing machine (Instron 8502, Instron, Norwood, MA, United States). Then, a monotonic ramp was applied in load control at a rate of 0.1 kN/s up to 1.0 kN, corresponding to a bending moment of 25 Nm. This value roughly simulates the bending moment occurring in the femur of a 60 kg active patient during daily activity (36). The vertical deflection of the mid cross-section was measured using an extensometer (Mod 2620-601 modified to increase the travel length to 10 mm, the accuracy of 0.01 mm; Instron, Norwood, MA, United States) fixed to the support frame. Each femur was tested alternating medio-lateral – generating tension on the lateral side to determine the coronal stiffness – and postero-anterior – generating a tension on the anterior side to determine the sagittal stiffness – load to avoid specimen conditioning and assess test repeatability, and estimated through the coefficient of variation. Five test repetitions were performed for each test configuration.

#### Torsional stiffness

All femora underwent torsional testing allowing free motion of the femur in the transversal plane. Before testing, each femur was then oriented vertically in an upside-down position using a three-degree of freedom clamping device. The femoral head was embedded in acrylic resin into a metal holder, leaving 150 mm between the mould surface and the midpoint of the femoral biomechanical length. The metal holder was fixed to a rail bearing and mounted orthogonally to the actuator of a biaxial testing machine (Minibionix 858 biaxial, MTS System Corp., Eden Prairie, MN, United States). The femoral axis was aligned with the load cell axis. Finally, the distal condyles were embedded in acrylic resin into a metal holder mounted onto the load cell, leaving a free length of 300 mm of the femur (Figure 1B).

A pretorque of 1 Nm was applied to the femur. Then, a monotonic ramp was applied in torque control at a rate of 3 Nm/s up to 25 Nm, i.e., 0.042 BWm (37), except for the end-to-end reconstruction, where the maximum torque was limited to 14 Nm to avoid bone plate yielding. During torsion testing, the axial channel was set at 0.01 kN in load control to avoid undesired compressive axial load during torsion. The twist angle, hereinafter referred to as rotation, was monitored during testing. As in the previous series, each femur was tested alternating intra- and extra-rotation to determine the intra- and extra-torsional stiffness. This approach avoided specimen conditioning and allowed to assess the test repeatability, calculated through the coefficient of variation. Five test repetitions were performed for each test configuration.

### Data processing and statistical analysis

The slope, i.e., the stiffness, of each load-displacement or torque-rotation curve was calculated to determine the slope of the best-fit line for each dataset. An iterative algorithm, which essentially consisted in discharging the first pair of load and displacement values of the dataset and repeating the fitting process, was performed until the coefficient of determination (R2) of the line of best fit reached the value of 0.995. Therefore, the non-linear response (if any) of the intercalary reconstruction at very-low load values was neglected.

The differences, in terms of bending or torsional stiffness, between different intercalary reconstructions were evaluated using the Friedman test. A non-parametric multiple comparison test (Conover’s test), based on the median rank differences of the groups, was used to analyse stiffness values determined for the three intercalary reconstructions stabilised with bone plates differing in junction geometry (38).

## Results

**FIGURE 3:**
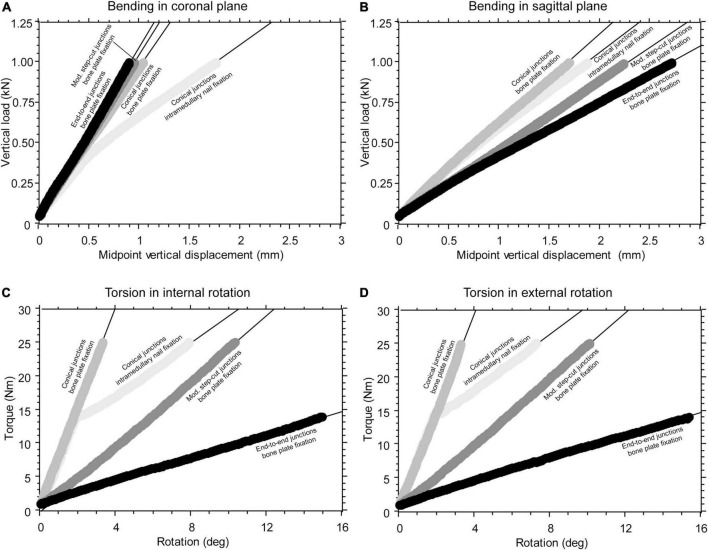
**(A)** Example of force-displacement curve collected during construct bending in the coronal plane generating tension on the lateral side. **(B)** Example of a force-displacement curve collected during construct bending in the sagittal plane, generating tension on the anterior side. **(C)** Example of a torque-rotation curve collected during construct torsion, causing intra-rotation of the proximal femur. **(D)** Example of a torque-rotation curve collected during construct torsion, causing extra-rotation of the proximal femur.

A total of 75 monotonic tests were carried out. The mechanical response to bending or torsional load of the intercalary reconstructions, including different junction geometries, was extremely linear (Figures 3A–D). The only exception was the intercalary reconstruction fixed by intramedullary nailing, showing a slightly decreasing slope with increasing bending moment (Figures 3A,B) or even a bilinear behaviour under torsional load (Figures 3C,D). In this latter loading condition, the construct stiffness changed when the transition from sticking to slipping occurred in the distal taper junction due to the increasing torsional load (Supplementary Video 1). The lowest values of the construct stiffness determined for this configuration were used in the analysis of data. The average repeatability was 3.8 and 3.4% for the bending and torsional test, respectively.

### Intercalary reconstructions stabilised with bone plate differing in junction geometry

**FIGURE 4:**
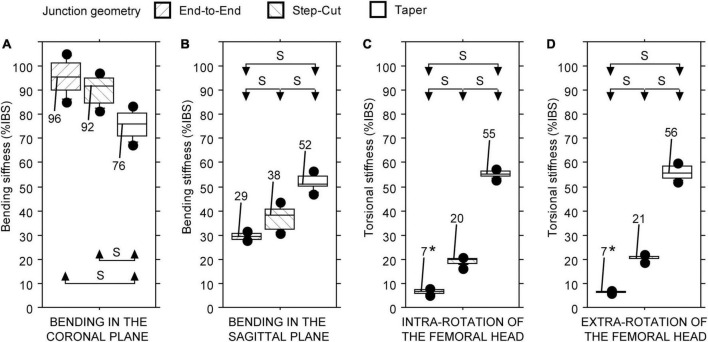
Bending and torsional stiffness, expressed as a percentage of intact host bone stiffness (%IBS) determined for the intercalary reconstruction with bone plate fixation differing in junction geometry. **(A)** Bending stiffness in the coronal plane. **(B)** Bending stiffness in the sagittal plane. **(C)** Torsional stiffness in intra-rotation direction. **(D)** Torsional stiffness in extra-rotation direction (S = statistically significant difference; * = test limited to 14 Nm).

Statistically significant differences in stiffness values of the three intercalary reconstructions were found regardless of the testing configuration (Friedman test: *P* < 0.01 in all four cases). The junction geometry impacted the intercalary reconstruction stiffness in different ways. The intercalary reconstruction with the end-to-end or modified step-cut junctions showed a bending stiffness in the coronal plane significantly higher compared with the intercalary reconstruction with taper junctions (Figure 4A). No significant differences were found between the other two intercalary reconstructions (Figure 4A). Conversely, the intercalary reconstruction with end-to-end junctions showed a significantly lower bending stiffness in the sagittal plane and an intra- and extra-torsional stiffness compared with the other two intercalary reconstructions (Figures 4B–D). The intercalary reconstruction with taper junctions showed a significantly higher bending stiffness in the sagittal plane and an intra- and extra-torsional stiffness compared with the intercalary reconstruction with modified step-cut junctions (Figures 4B–D).

### Intercalary reconstruction with taper junctions differing in the type of fixation

**FIGURE 5:**
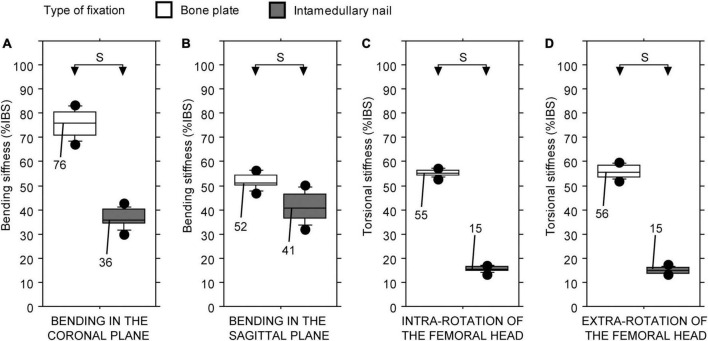
Bending and torsional stiffness, expressed as a percentage of intact host bone stiffness (%IBS) determined for the intercalary reconstruction with the taper junctions differing in the type of fixation. **(A)** Bending stiffness in the coronal plane. **(B)** Bending stiffness in the sagittal plane. **(C)** Torsional stiffness in intra-rotation direction. **(D)** Torsional stiffness in extra-rotation direction (S, statistically significant difference).

Statistically significant differences in stiffness values of the two intercalary reconstructions were found regardless of the testing configuration (Friedman test: *P* < 0.01 in all four cases). The type of fixation consistently impacted the intercalary reconstruction stiffness: the fixation with bone plate provided higher construct stiffness than the intramedullary nail, regardless of the loading configuration (Figures 5A–D).

## Discussion

The aim of this study was to compare the bending and torsional stiffness of different intercalary reconstructions in synthetic femora. The chosen experimental procedure is suitable to provide comparative data, although with some of the following limitations: (i) the use of synthetic femora neglects any tissue and/or anatomical difference among patients; (ii) intercalary reconstructions were performed under optimal conditions, i.e., achieving a perfect match at the host-graft interface and neglecting the presence of surrounding soft tissues; (iii) monotonic mechanical tests were performed aiming at investigating the postoperative mechanical condition and neglecting mid- and long-term changes in stiffness of the intercalary reconstructions due to biological processes.

Nevertheless, this proof-of-concept study provides experimental evidence that the host-graft junction has a significant impact on the bending or torsional stiffness of the intercalary reconstruction. The taper junction, fixed with the bone plate, works on the same principle of a one-piece setscrew collar combined with lateral reinforcement. This solution increases the load-carrying capacity of the host-graft junction, especially the torsional one. In fact, the largest improvements were found in torsional stiffness values. Replacing the bone plate with an intramedullary nail changes the load transfer mechanism, especially the torsional one (39). In fact, friction at the host-graft interface and torsional stiffness of the intramedullary nail determine the transition from sticking to slipping and the subsequent elastic response of the reconstruction. Since longitudinal compaction, presence of external axial loads, bone-to-bone coefficient of friction [which differs from synthetic bone-to-synthetic bone (40, 41)], and cross-sectional shape and area of the intramedullary nail all impact the torsional load, determining the aforementioned transition, the reported values cannot be used to set a threshold for clinical applications. However, surgeons who opt for intramedullary nail fixation must be aware that allowing early (i.e., before evidence of callus formation) full weight-bearing causes torsional loads (e.g., sitting on/raising from a chair, navigating stairs, and running) (42, 43) may lead to rotational slippage at the host-graft interface, which could increase the risk of delayed healing or non-union (44, 45).

Bending stiffness can be divided into sagittal and coronal bending stiffness. Regardless of the type of fixation, replacing end-to-end or step-cut junctions with conical ones, the sagittal bending stiffness of the intercalary reconstruction is increased. This is hardly surprising, since, under sagittal bending moments, both bone plate and intramedullary nail are roughly aligned with the neutral axis, and therefore, their contribution to the second moment of inertia of the construct is small. Under this condition, the conical junction contributes significantly to the sagittal bending stiffness by ensuring load transfer further away from the neutral axis compared with both fixation types. This argument still applies to the intercalary construction that is fixed using the intramedullary nail when the construct undergoes coronal bending moments. Conversely, the bone plate is distant, on the tension side, from the neutral axis during the coronal bending moment. Therefore, it counteracts the lateral tensile load while the host-graft interface undergoes non-uniform compressive stress, creating a pivot point at the far cortex. Replacing the end-to-end with a step-cut junction determines a small medial shift of the pivot point with negligible effects on bending stiffness. Conversely, the taper junction changes the interface geometry leading to a more marked medial shift of the pivot point, resulting in a decrease in bending stiffness. However, the coronal bending stiffness still remains high, the reconstruction providing a median stiffness value of 76% IBS.

It must be acknowledged that the aforementioned load transfer mechanisms are greatly affected by the stiffness of the fixation devices: the stronger the fixation device, the stiffer the intercalary reconstruction (46). Increasing the size of the fixation device can significantly change the present bending and torsional stiffness values, but with an associated increased risk of stress shielding (47, 48). Stress shielding within bone tissue at the level of the intercalary reconstruction might determine negative bone remodelling, especially when reconstruction is combined with a vascularised fibular autograft (49–51). From a biomechanical point of view, this study confirms the advantages of an inherent stable host-graft connection (52–54), i.e., an alternative way to increase the stiffness of the intercalary reconstruction can be by changing the load transfer mechanisms through different host-graft junctions.

Regardless of the load transfer mechanism, the taper junction allows to achieve an intercalary reconstruction with a more homogeneous response to different loading conditions, which should ensure a more stable reconstruction despite the complex loading condition it undergoes *in vivo*. It could be argued that the need to reaming both stump and graft ends further complicates an already complex surgery. However, the use of conical reamers would make the additional step easier (55), ensuring proper tapering of the host bone and graft ends. Tapering the host bone and graft ends provides an increase in contact surface by approximately 220 and 480% compared with that found in modified step-cut and end-to-end shapes, respectively. A greater contact area between the graft and viable host bone tissue should also facilitate cell colonisation of the graft, as suggested by early clinical results achieved with different intercalary reconstructions or telescope allograft techniques (22, 52, 56, 57).

## Conclusion

The findings of this study show that intercalary reconstructions with taper junctions, fixed with a low-stiffness bone plate, demonstrate, under four different loading conditions, greater homogeneity and, in three out of four conditions, higher mechanical stiffness compared with intercalary reconstruction with end-to-end or modified step-cut junctions. Replacing the bone plate with an intramedullary nail decreases the stiffness of the intercalary reconstruction, especially in terms of torsional stability. Regardless of the type of fixation, tapering of the stump and graft ends increases the contact between the graft and viable host bone tissue, with a potential beneficial effect on cellular colonisation of the graft ends.

The taper junction with low-stiffness fixation could be a useful alternative to achieve intercalary reconstructions with stiff responses to different loading conditions and reduce the risk of late cortical atrophy.

## Data availability statement

The raw data supporting the conclusions of this article will be made available by the corresponding author upon request, without undue reservation.

## Author contributions

MBa and MM contributed to the conception and design of the study and supervised all project tasks. MP performed the surgical tasks. PE, MBl, and RF prepared specimens, performed mechanical tests, and collected and analysed the data. MBa wrote the first draft of the manuscript. MM wrote clinical sections of the manuscript. All authors contributed to manuscript revision, read, and approved the submitted version.
